# Separating triplet exciton diffusion from triplet–triplet annihilation by the introduction of a mediator†

**DOI:** 10.1039/d4sc07004f

**Published:** 2024-12-09

**Authors:** Andrew J. Carrod, Anton M. Berghuis, Vishnu Nair Gopalakrishnan, Andrew Monkman, Andrew Danos, Karl Börjesson

**Affiliations:** a University of Gothenburg, Department of Chemistry and Molecular Biology Medicinaregatan 7B 41390 Gothenburg Sweden karl.borjesson@gu.se; b Dutch Institute for Fundamental Energy Research P.O. Box 6336 5600 HH Eindhoven The Netherlands; c Department of Physics, Durham University South Road Durham DH13LE UK; d School of Physical and Chemical Sciences, Queen Mary University of London London E1 4NS UK

## Abstract

Triplet–triplet annihilation photon upconversion (TTA-UC) combines the energy of two photons to provide one of higher energy that can be used to drive photochemical or photophysical processes. TTA-UC proceeds at high efficiencies in dilute solution, but in solid state the efficiency drastically reduces. This is because exciton diffusion, compared to molecular diffusion in solid annihilator films, suffers concentration induced quenching, undermining efficient emission. Here, we provide a method to decouple the triplet exciton diffusion and the annihilation processes using an exciton transporting mediator as host. At low exciton densities emission occurs from the annihilator, while at higher exciton intensities TTA and emission from the mediator is observed. The low concentration of the annihilator dopant gives evidence for a hetero-TTA mechanism being active, *i.e.* annihilation occurring between the mediator and an annihilator molecule. Monte-Carlo simulations qualitatively reproduced the experimental results and give a direction for future optimization. This work hence demonstrates successful separation of exciton diffusion from annihilation by the introduction of a triplet mediator host, and with this approach support the development of highly efficient solid-state TTA-UC materials.

## Abstract 2

The functions of triplet diffusion and annihilation are seperated into two different molecules by using triplet triplet annihilation between two diffrenet molecular species, allowing for photon upconversion at low annihilator concentrations.

## Graphical abstract

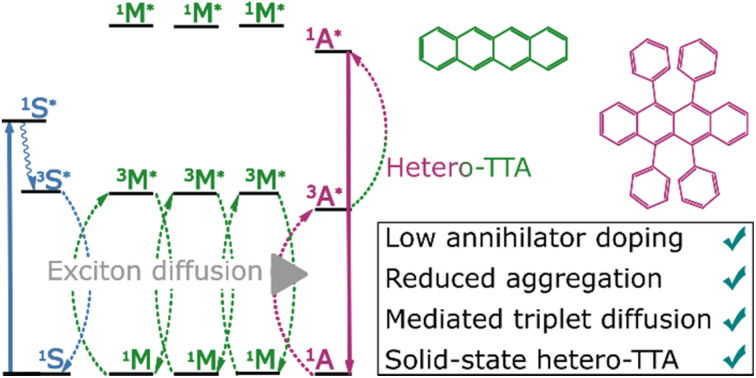

## Introduction

Triplet–triplet annihilation upconversion (TTA-UC) is a process which converts two low energy photons to one higher-energy photon through a sequence of energy-transfer steps.^1–3^ Commonly carried out in solution,^4–10^ TTA-UC utilizes a sensitizer and annihilator pair. The sensitizer absorbs low energy photons, undergoes intersystem crossing to form triplet excitons, and transfers these to the annihilator. Annihilator molecules in their excited triplet states can subsequently come into close contact, and the triplet states can recombine through TTA. This yields one annihilator in its excited singlet state and the other in the ground state. Emission then occurs, as it would by direct excitation of the singlet state of the annihilator. TTA-UC has been applied to drive processes using low energy photons in a variety of fields, from catalysis to biosensing.^11–16^ However, no application has received as much interest as photovoltaics, in which the process of TTA-UC could enable harvesting of below bandgap-photons, thus augmenting the theoretical maximum efficiencies of solar harvesting devices.^17–20^

For TTA-UC to become technologically relevant within solar harvesting, solid-state TTA-UC systems need to be developed. Unfortunately, whilst there have been many reports on highly efficient TTA-UC systems in solution, the development of efficient solid-state upconverters remains elusive.^21^ Some challenges have included material stability and oxygen sensitivity.^22–24^ However, the biggest problem associated with going from solution to solid state is replacing molecular diffusion by exciton diffusion. Exciton hopping requires molecular orbital overlap, and therefore annihilator concentrations approaching the neat state, which in turn results in significant concentration-induced quenching of the annihilator singlet state.^25–27^ Thus, a pressing fundamental matter in solid-state TTA-UC films is achieving efficient excited-state energy transfer between species, whilst maintaining the annihilator's favorable photophysical properties.^28–30^ The inclusion of a second emitter at dopant concentrations has been investigated to mitigate this issue,^31–34^ although no high performance systems have so far been made. Furthermore, efficient quasi-solid-state systems have been made in solvated pores or rubbery polymeric materials, which allow for molecular diffusion.^35–39^ Whilst each of these methods achieves success in improving upconversion yields, they create their own challenges such as keeping the films or pores stable to drying over time. Therefore, if solid state sensitizer–annihilator films could be fabricated with efficient energy transfer and without concentration-based emission quenching, this would constitute a significant step towards efficient solid state TTA-UC.

**Fig. 1:**
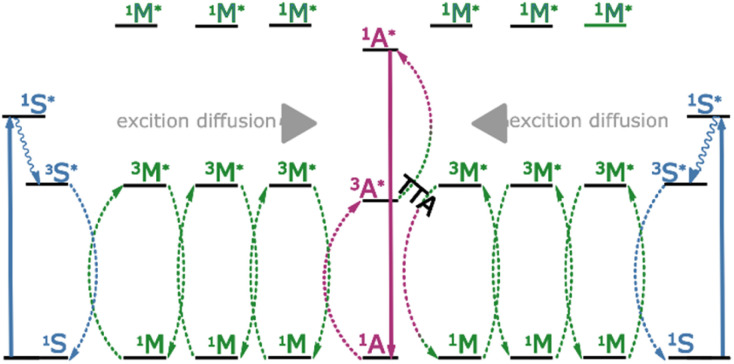
Schematic diagram of the energy transfer pathways and the photon upconversion mechanism in this work. Photon absorption and ISC occurs on the sensitiser (S), and triplet energy transfer to the mediator (M) proceeds with high efficiency. Exciton diffusion between mediators is rapid and occurs over long distances, resulting in hetero-TTA at the annihilator (A) sites.

Hetero-TTA, so called because it involves annihilation between two excited molecules of different compounds, has so far only been confirmed in solution.^40–42^ However, hetero-TTA has also been suggested to function in the solid state. In this context one annihilator may additionally act as a dispersive host for the other, and thus overcome aggregation effects.^43^ In this work we investigate TTA in the solid state, and propose a mixed-material platform and scheme of energy transfer steps that allows triplet energy transfer to be separated from upconversion and emission. To do so we propose the utilization of a hetero-TTA based mechanism, specifically using a low concentration of annihilator molecules (and sensitizers) within a mediator matrix (Fig. 1).

Our proposed mechanism relies on an initial triplet energy transfer step from sensitizer to mediator (^3^S* → ^3^M*). This is followed by triplet exciton diffusion in the mediator matrix (^3^M* → ^3^M*). Finally, triplet energy transfer from the mediator to the annihilator occurs (^3^M* → ^3^A*), with the low triplet energy of the annihilator effectively acting as a sink (and spatially confining trap) for the triplet excitons. A second sequence following the same steps and terminating at a mediator in the vicinity of the excited annihilator may then produce a singlet excited state on the annihilator through hetero-TTA between the mediator and the annihilator (^3^M* + ^3^A* → ^1^A* + ^1^M). This mechanism allows efficient funneling of triplet excitons to annihilator sites, to produce the desired excited state species (^1^A*) without molecular diffusion. We herein investigate each step in the energy transfer series experimentally, using time resolved and steady state emission spectroscopy coupled with Monte-Carlo simulations. We find that even when small percentages of an annihilator are doped into an appropriate system, hetero-TTA becomes the predominant upconversion mechanism that can be exploited in solid state TTA-UC. This allows for the separation of the triplet energy transfer from the annihilation process, allowing them to occur with efficiencies determined by different molecules, thus enabling individual molecular specialization and optimization.

## Results and discussion

The first challenge in designing a system energetically resembling that in Fig. 1 is to find molecules with compatible excited state energies. Rubrene was selected as the annihilator. Its highly advantageous upconversion properties include a red emission matching the bandgap of perovskite solar cells, a high fluorescence quantum yield, and a moderate to high spin statistical factor.^32,44,45^ However, rubrene has been the subject of much disappointment when attempting to switch from solution to solid state. Unlike in solvated samples, aggregation induced quenching and inner filter effects become readily apparent in neat rubrene films (Fig. S5†).^46–48^ A 30 fold increase in fluorescence quantum yield is observed on returning to dilute conditions (doped films), although mitigation of quenching in this way is accompanied by a reduction in exciton transport, which is detrimental for TTA.^32^

To spatially separate rubrene molecules while maintaining efficient energy transfer, a mediator molecule that facilitates triplet energy transfer (TET) is required. Properties supporting TET include a triplet energy level lying between the sensitizer and rubrene, an S_1_ state being high in energy (2E_T1_ < E_S1_, to suppress host–host TTA), and a large orbital overlap in the solid state. Within the context of TTA-UC and singlet fission, the photophysical properties and exciton dynamics of neat tetracene films have been widely studied.^49–52^ It efficiently forms two triplet states from its initially excited singlet state even in thin films.^53^ Although TTA-UC is still possible (2E_T1_ ≈ E_S1_), the equilibrium favors singlet fission. Whilst rubrene can also perform both TTA-UC and singlet fission, its energetics also favor the former while the latter will be impossible in sufficiently diluted films. In this application, singlet fission within tetracene is not actively exploited, and largely avoided by choice of excitation wavelength. Instead, the T_1_ energy alignment helps to enhance the triplet exciton diffusion in a tetracene matrix, wherein rubrene can then be dispersed at low concentration.

### Investigation of individual energy transfer processes

**Fig. 2:**
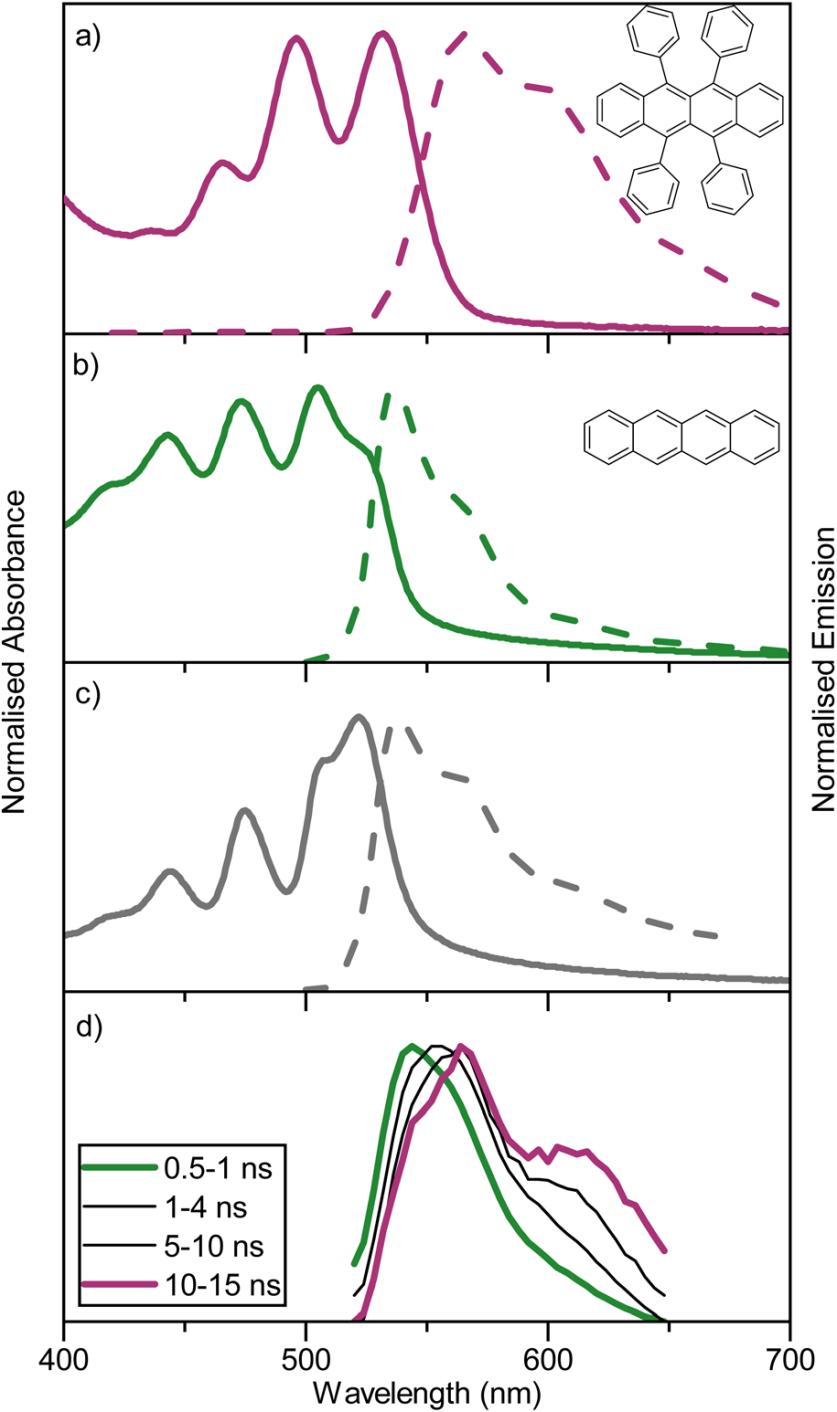
Absorption and steady-state emission spectra for evaporated films of (a) rubrene, (b) tetracene, and (c) 5% rubrene in tetracene (*l*_exc_ = 475 nm), taken as 100 nm thin films on quartz substrates fabricated through thermal evaporation. (d) Normalised TRES spectra of a thermally evaporated film (100 nm thickness on quartz) containing 5 vol% rubrene in tetracene, integration times after the excitation pulse are indicated.

For an annihilator–mediator pair to function in the context of TTA-UC, it is important that the S_1_ energy of the annihilator is smaller compared to that of the mediator to avoid reabsorption of the upconverted emission. To confirm this property, amorphous neat films of rubrene, tetracene, and rubrene-doped tetracene (5 vol%) were made by thermal evaporation (so as to ensure homogenous doping and avoid thick crystalline regions associated with solution processing).^54^ The fabricated films gave absorption and emission characteristics (Fig. 2a–c) similar to that previously reported.^55^ Rubrene show an absorption maxima at 532 nm and emission maxima at 566 nm, while tetracene has absorption and emission maxima of 505 and 538 nm, respectively. The blend containing 5 vol% rubrene shows spectra resembling tetracene with matching absorption and emission wavelengths, unsurprising given its composition. Furthermore, GIWAXS indicate a retained although slightly disturbed film packing in the blended film compared to a neat tetracene film (Fig. S6†). Supporting a picture of a non-phase separated system. These spectra indicate that singlet state energy transfer from tetracene to rubrene is inefficient, as was expected based on the short Förster distance (1.6 nm). To explore energy relaxation in the blended system more carefully, time resolved emission spectroscopy was performed (Fig. 2d). At short timescales, the emission is tetracene-like. However, at longer delay times, the emission envelope becomes more and more rubrene-like, which is as expected regarding the difference in lifetimes of neat films of the two molecules (1 *vs.* 3 ns). Together these results suggest that detrimental back energy transfer from the annihilator to the mediator is not a dominant relaxation pathway. Instead, the tetracene-like steady-state emission is explained by the high concentration (and thus absorption) of tetracene in the blend, and the energy diagram can be written without substantial transfer from the tetracene S_1_ state to the rubrene S_1_ state (as in Fig. 1).

**Fig. 3:**
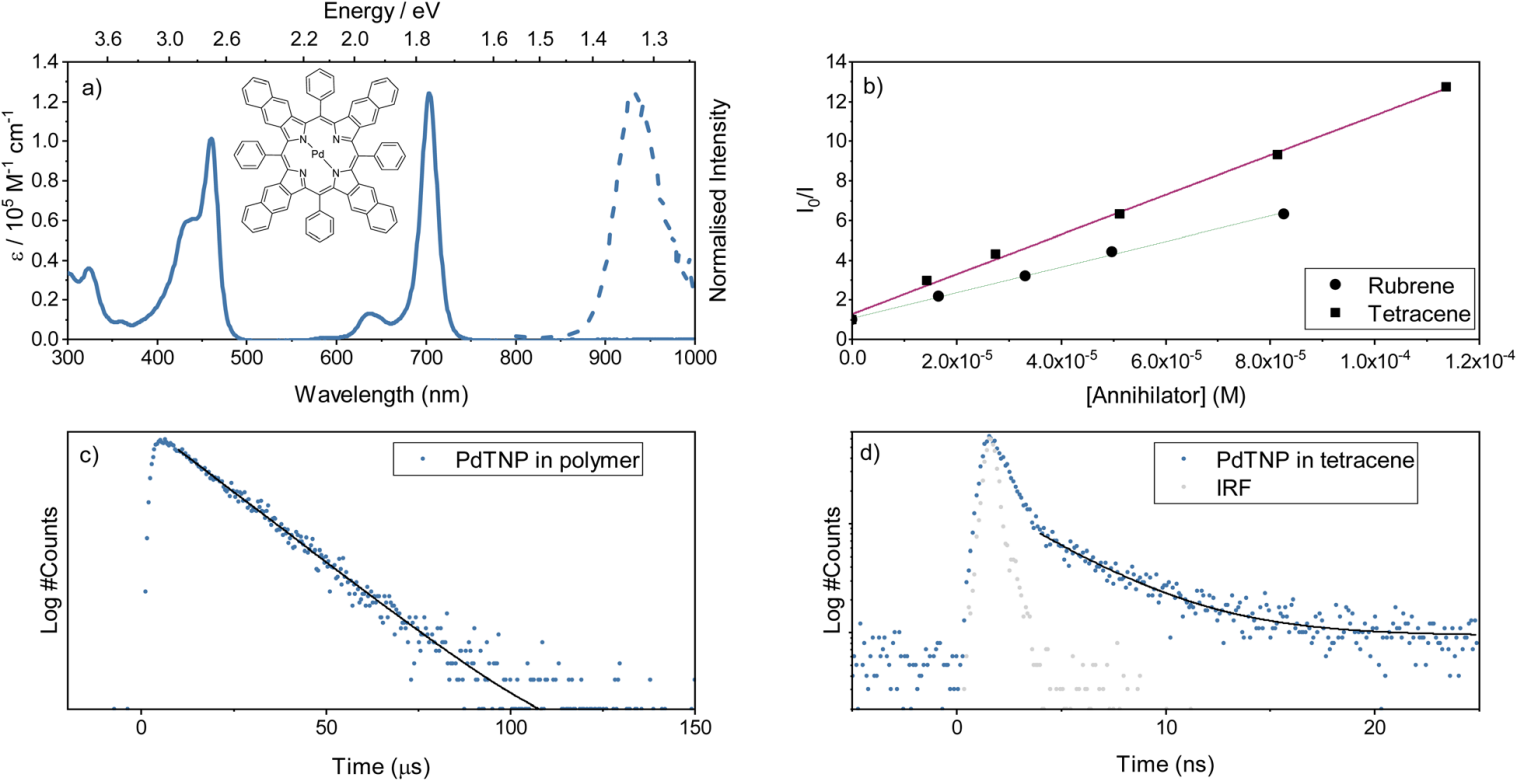
(a) Absorbance (solid) and emission (dashed, *l*_exc_ = 705 nm) spectra collected for the porphyrin, PdTNP (5 mM in toluene). (b) Stern–Volmer plot for the dynamic quenching of PdTNP by both mediator and annihilator (*l*_em_ = 930 nm, toluene). (c) Phosphorescence decay of 1% PdTNP in polystyrene (processed by spin coating) using the multichannel scaling technique (*l*_exc_ = 705 nm). (d) Phosphorescence decay of 1% PdTNP in tetracene using the TCSPC technique (*l*_exc_ = 475 nm, *l*_em_ = 930 nm, 500 nm on quartz, blue dots), grey dots indicate the measured IRF.

Separate from the annihilator and mediator, the sensitizer must capture incident low-energy photons and efficiently transfer energy to the mediator triplet states. The porphyrin PdTNP was used here to fulfil this function (Fig. 3a). This class of pi-expanded porphyrins display strong absorption bands stretching into the far-red and NIR spectral region with long-lived luminescence arising from the triplet state (Table S2,† and Fig. 3a). In solution, PdTNP show a very strong absorption from the 0–0 transition of its S_1_ state, centered around 700 nm (1.77 eV). Phosphorescence is long-lived, with a lifetime of 34 ms (Fig. S7†) and is centered at 930 nm (1.33 eV), which is nearly isoenergetic with the first triplet excited state of tetracene (1.25 eV)^52^ and above that of rubrene (1.14 eV).^32^ Thus, the choice of sensitizer is energetically compatible with the mediator. To determine if the PdTNP sensitizer transfers energy efficiently to both tetracene and rubrene, a Stern–Volmer analysis was performed using steady state conditions in toluene (Fig. 3b and S8†). Near diffusion-limited quenching constants of 2.9 and 1.9 × 10^9^ M^−1^ were observed for tetracene and rubrene, respectively (Fig. 3b). The slightly increased quenching constant for tetracene compared to rubrene can be explained by the phenyl groups, providing shielding against triplet energy transfer occurring and/or reducing the rate of diffusion due to the increased size of the molecule.

Whilst triplet energy transfer from sensitizer to mediator was confirmed in solution, this does not always translate directly into the solid state. Phosphorescence lifetimes, arising from the PdTNP sensitizer, (1%) were collected when dispersed in polystyrene or tetracene (Fig. 3c and d). When dispersed in polystyrene the lifetime of PdTNP was 15 ms, slightly shorter than that collected in toluene solution. At the same concentration in tetracene the emission lifetime falls to 4 ns, signifying an extremely efficient energy transfer and quenching of sensitizer triplet excitons. This result indicates that the energy levels between PdTNP and tetracene remain favorably aligned in the solid state, and that a large orbital overlap exists between the two molecules. The triplet energy transfer step (^3^S* → ^3^M*) in Fig. 1 therefore proceeds with high efficiency and is not considered a limiting step in the TTA-UC mechanism. In summary, the PdTNP–tetracene–rubrene trimolecular system has the correct energetics to form the envisioned sensitizer–mediator–annihilator energy funneling scheme.

### Upconversion in binary blends

**Fig. 4:**
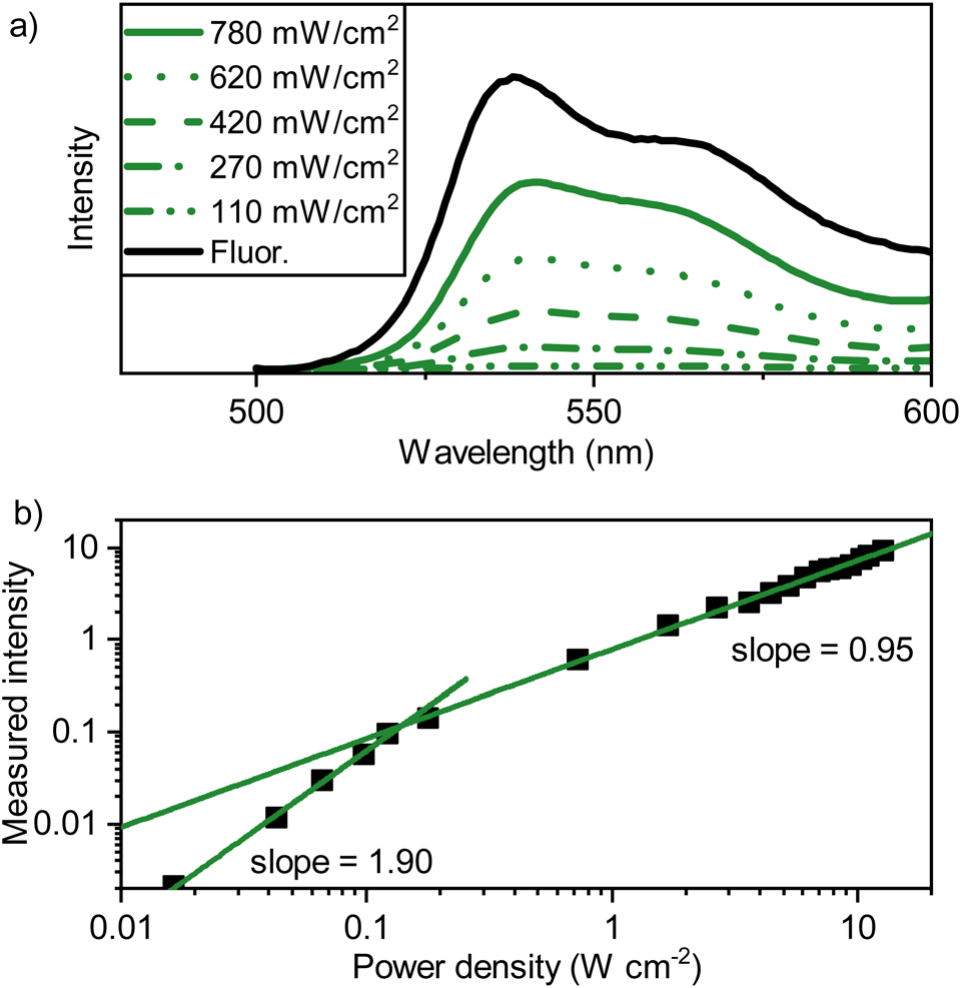
(a) Emission spectra of a PdTNP–tetracene film (evaporated; 500 nm on quartz, 1% PdTNP, green lines) with incident power of the laser beam indicated (*l*_exc_ = 690 nm, CW, *Ø* = 2 mm). In black the conventional fluorescence spectrum (*l*_exc_ = 475 nm) of a neat tetracene film is shown, (b) power density dependence of the PdTNP–tetracene thin film emission, excited at 690 nm.

To be able to rationalize the effect of rubrene as a triplet sink, binary sensitizer–tetracene blends were first spectroscopically investigated. Starting with the UV-Visible absorption spectrum (Fig. S9†), strong tetracene absorption with vibronic peaks below 540 nm were observed, as was the presence of the sensitizer absorption at 713 nm, a slight bathochromic shift from the value observed in toluene solution. The emission was also investigated (exciting the PdTNP at 690 nm), specifically that of upconverted photons arising from the recombination of triplet-excitons within the tetracene mediator (Fig. 4a). The emission profile is comparable to that observed for the fluorescence in neat tetracene films (Fig. 2a), albeit 3 nm shifted to 541 nm. This shift is likely explained by inner filter effects. Thus, triplet excitons are formed, can diffuse, and even recombine within the tetracene matrix.

The upconverted emission shows a power dependency typical of TTA-UC with an *I*_th_ of 0.14 W cm^−2^ (Fig. 4b), with no changes observed in the peak shape throughout all measurement points. These results indicate that triplet-excitons are mobile within the PdTNP–tetracene blend. Although, whilst ^3^M* → ^3^M* is an active process, the formation of upconverted photons nonetheless prevents tetracene from being the ideal matrix. Ideally, the matrix should be TTA-inactive, with E_S1_ ≫ 2E_T1_, thus allowing TTA to occur only on the dilute annihilator sites.

### Upconversion in ternary blends

**Fig. 5:**
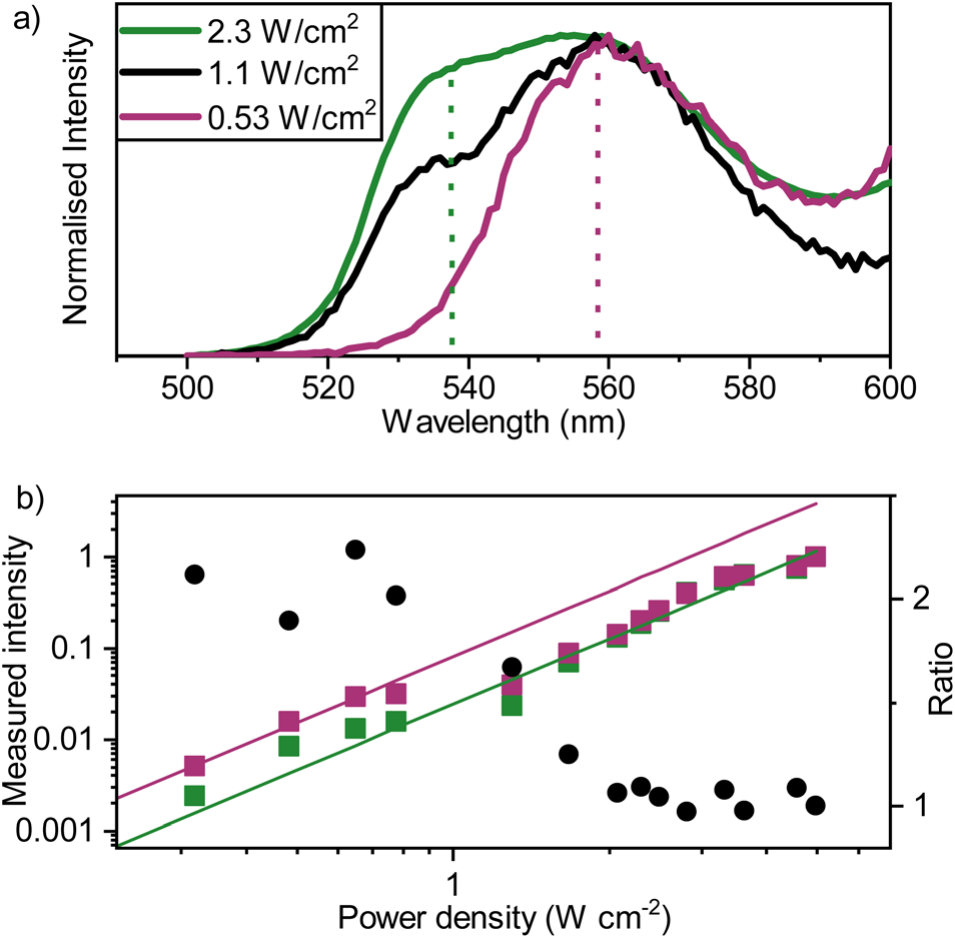
(a) Normalized emission spectra of PdTNP–tetracene–rubrene films (evaporated; 500 nm on quartz, 1% PdTNP and 1% rubrene) with incident power of the laser beam indicated (*l* = 690 nm, CW, *Ø* = 2 mm). The vertical dashed lines indicate the fluorescence maximum of neat films of tetracene (green) and rubrene (purple), indicating the likely emitter species. (b) Power density dependence of the ternary blended thin film emission measured at 540 nm (green squares) and 560 nm (purple squares) using 4 nm slight widths, together with the ratio between the two signals (black circles).

The final process in the proposed upconversion system is hetero-TTA (Fig. 1). To explore this process, sensitizer–mediator–annihilator blends were fabricated. These films show similar absorbance features to those of the binary blends (Fig. S9†). However, the upconversion emission profile arising from recombination of triplet-excitons within the ternary blends differed (Fig. 5a). The effect of the excitation power on the emission envelope has previously been used to detect excimers consisting of an excited state triplet and ground state singlet.^56,57^ Here it is instead used to favor one annihilation pathway over another. The emission profile itself is significantly shifted compared to that of the sensitizer–mediator blend (560 nm *vs.* 538 nm). At low excitation intensities, the spectral envelope indeed resembles that of rubrene. From the low percentage of rubrene present, we propose that this must be a result of hetero-TTA events, as the likelihood of two rubrene molecules being neighbors to form a homogenous triplet pair (or aggregate) is small (estimated average separation ∼4 nm in films). Increasing the excitation intensity, a second peak becomes more intense, at an energy corresponding well to where tetracene emission would be expected (538 nm, 2.31 eV). This trend indicates the increased prevalence of homo-TTA between tetracene molecules at higher exciton densities. These experiments were duplicated with non-sequential power values to ensure that the effect is consistent and does not arise from bleaching of rubrene or degradation of the tetracene matrix. Good agreement was found between runs (Fig. S10†).

To assess the effect of hetero-TTA on the upconverted emission efficiency, excitation power dependent measurements were further analyzed. At high fluence only tetracene emission is evident (Fig. 5a), indicating that only homo-TTA is active. Emission extracted at the rubrene and tetracene maxima were then normalized at the highest excitation density to facilitate comparison. Fig. 5b show the power density dependence, extracted at 540 and 560 nm. Two lines, describing emission power dependence at low (hetero-TTA active, purple) and high (homo-TTA active, green) excitation densities, are shown. A clear shift in emission intensity is observed at an excitation power density of 1 W cm^−1^, and before and after this emission intensity shift, the derivative of the curve is the same. Thus, the change in the curve is not due to a transition from different power regimes. Instead, it is exactly what we expect based on our simulations that include a hetero-TTA mechanism (*vide infra*). These lines therefore represent the relative upconversion quantum yield with system parameters kept constant (sensitizer concentration, crystal packing *etc.*). The relative upconversion quantum yield is larger at low fluence where hetero-TTA is active, showing a real benefit of our approach over binary blends.

The absolute upconversion quantum yield at high excitation fluence was low, around 6.4(±2.4) × 10^−5^% (working at high fluences was required for adequate signal to noise in sphere-based PLQY measurements). In comparison, the yield for the binary film was larger, 2.7(±0.2) × 10^−3^%, although still small in an absolute sense. The reason for the lower yield for the ternary blend is most likely the lower crystallinity of the tetracene matrix (as shown by GIWAXS; Fig. S6†), resulting in slower triplet diffusion and a difficulty in reaching the linear regime with our excitation source. The lower crystallinity was equally a determining factor when examining the upconverted emission lifetimes. The upconversion lifetime is expected to be dominated by the triplet lifetime of tetracene, which has been shown to be around 50 ns for polycrystalline tetracene and decrease with decreasing crystallinity.^58^ This is therefore in line with our measured values of 37 and 57 ns for the ternary and binary blends, respectively (Fig. S11†). In summary, these experiments suggest that hetero-TTA is active in these films, that the dominance of hetero or homo-annihilation events is excitation intensity dependent, and that the annihilator dopant is providing an enhanced emission yield at low excitation fluences.

### Monte-Carlo simulations

Reproducing experimental results of TTA-UC in solution is straightforward using rate equations. Such methodology is however not as easily applied in the solid state. To simulate the sensitizer–mediator–annihilator solid state system, inspiration was therefore taken from the field of organic electronics, where Monte-Carlo based methods have been applied to explain the role of exciton diffusion in excited state dynamics of organic molecules.^59–63^ Modelling the events at the level of individual excitations can give insight into how excited states diffuse and interact with each other, which cannot be achieved by the treatment of exciton populations as a density distribution governed by a set of coupled rate equations.

The goal of these Monte-Carlo simulations was to qualitatively reproduce the experimental findings, to get a more detailed understanding of the competition between hetero- and homo-TTA in the ternary blends, and to estimate the maximum potential benefit of an ideal mediator (which TTA-active tetracene was shown not to be) on the upconversion yield. Monte-Carlo simulations are based on time steps, where rate constants are translated to a probability for the photophysical transformations to happen during each time step. The time steps are then consecutively solved until equilibrium is reached, at which point the probability for an event, such as emission, can be integrated and evaluated.

**Fig. 6:**
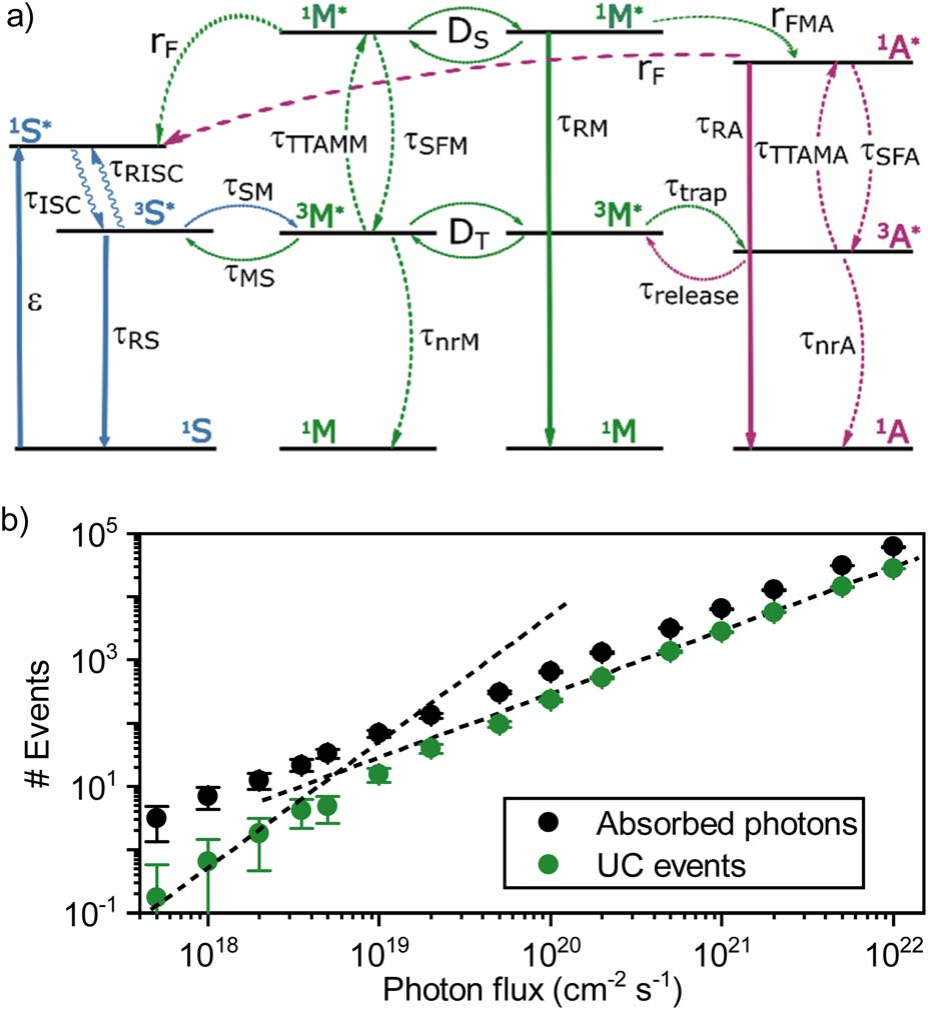
(a) Scheme of the rates used in the Monte-Carlo simulations. The values of rates are given in Table S1.† (b) Emission events as a function of the incident photon flux for a system of 1% sensitizers in a host matrix (tetracene). The two dashed lines have a slope of 1 and 2 as visual guides.

To test this approach in a TTA-UC setting, the mediator – sensitizer matrix was first explored. A 50 × 50 × 50 nm cube containing tetracene doped with 1% (mol mol^−1^) of PdTNP was constructed, and the effect of the excitation intensity on upconversion emission was evaluated. At this point, only interactions within the sensitizer, between the sensitizer and mediator, and between mediators were considered (blue and green arrows in Fig. 6a, see ESI for a full description including used rate constants, Table S1†). Fig. 6b shows the number of absorbed photons by the sensitizer and upconversion events by the mediator as a function of photon flux. The number of absorbed photons follows a linear dependence with the photon flux, which indicate that no saturation effects are affecting the analysis. The upconversion data on the other hand show a linear dependence at a high photon flux, but a quadratic dependence at a lower photon flux. The transition point between the linear and quadratic regime is around 4 × 10^18^ photons per cm^2^ per s, which translates to an excitation intensity of 1.2 W cm^−2^. This result is in qualitative agreement with the experiments although quantitatively the transition from quadratic to linear regime occurs at a density approximately one order of magnitude larger than in the experimental case (Fig. 4b). The larger value of the simulated *I*_th_ can be due to underestimates of loss rates and/or sensitizer concentration. These results nonetheless establish that simulating the *I*_th_ of TTA-UC in the solid state is possible by a Monto-Carlo based approach.

**Fig. 7:**
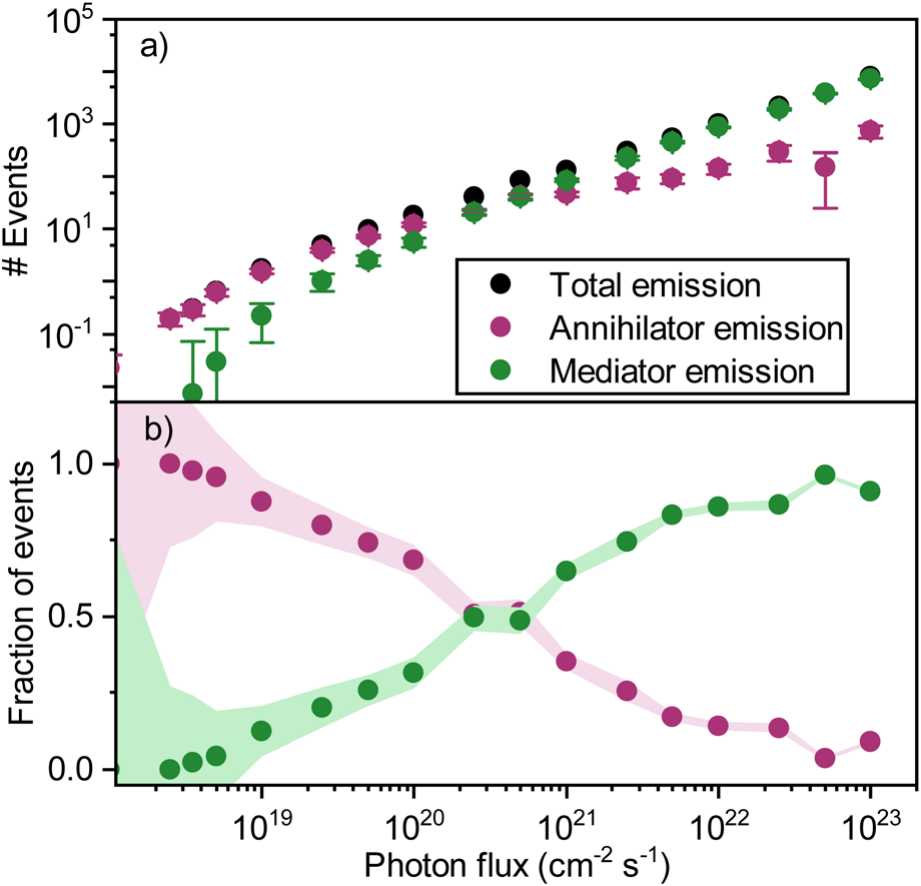
(a) Simulated emission events resulting in emission in a ternary blend with 1% sensitizers and 0.1% of annihilators in a host (mol mol^−1^). (b) Simulated fraction of upconversion events due to hetero-TTA (purple) and homo-TTA (green).

When adding the annihilator molecule at a suitably low dopant concentration to the model (purple arrows in Fig. 6a), there arise two possible routes for annihilation to occur. The first one is the previously discussed homo-TTA (mediator–mediator annihilation), now accompanied by annihilation between a triplet mediator and a triplet annihilator molecule (hetero-TTA). The result of the additional pathway on the simulations is plotted in Fig. 7a. At low photon fluxes, the density of triplet states is small, resulting in a low rate of homo-TTA (and a dominant hetero-TTA channel) and therefore a maximized triplet lifetime. The time for a mediator triplet to diffuse and find an annihilator (the energy sink) is also therefore long. When increasing the photon flux, the concentration of mediator triplet states increases, enhancing the rate of homo-TTA, and reducing the triplet lifetime. The likelihood of the triplet exciton diffusing far enough to encounter a dilute rubrene site without first undergoing homo-TTA is reduced, explaining the levelling-off (Fig. 7a) and relative decline (Fig. 7b) of the hetero-TTA channel with increased photon flux, and concomitant rise of the homo-TTA channel. The regime change where homo-TTA becomes the majority annihilation mechanism occurs at a photon flux of about 10^19^ photons per cm^2^ per s, which translates to a power density of around 3 W cm^−1^ (Fig. 7b).

In the binary blend, a dominant loss mechanism was found to be quenching of upconverted singlet excitons in the mediator molecules by Förster type energy transfer to the sensitizer.^64^ A large potential advantage of an envisioned ideal ternary blend is that the upconverted singlet state is energetically trapped at the annihilator molecule. Therefore, these singlets do not diffuse, which together with the low concentration of both sensitizer and annihilator molecules result in a smaller chance to come into energy transfer distance to the sensitizer (minimizing FRET). The propensity of the singlet state to be quenched therefore reduces, which can be seen in the simulations as a photon flux dependency on the energy transfer efficiency (Fig. S12†).

The largest benefit of introducing a ternary blend, *i.e.* separating the processes of triplet energy transfer and annihilation, is so far not considered in the modelling. This is because tetracene is a non-ideal mediator, with a singlet excited state that significantly contributes to the photophysics in the ternary blend. It is however interesting to explore computationally what would happen if homo-TTA in the mediator is deactivated. Experimentally this would require the development of molecules with a very large separation between the S_1_ and T_1_ states, efforts towards which we hope will be stimulated by this work. It is however trivial to turn off this process in the Monte-Carlo simulations, and by so doing predict the enhancement that an ideal mediator molecule could provide compared to non-ideal tetracene. When so doing, the number of hetero-TTA events does not level off with the photon flux anymore (Fig. S13a†). They are instead on the same level as the total number of events when homo-TTA is active. Furthermore, considering that the fluorescence quantum yield of the annihilator is much higher when dispersed in a high bandgap host (Fig. S5†), the total number of emission events drastically increases (Fig. S13b†).

**Fig. 8:**
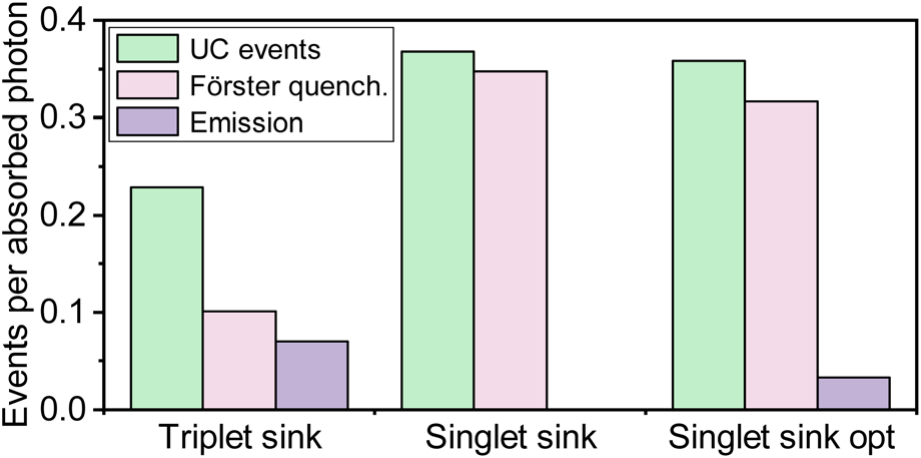
Comparison of the singlet sink approach and the proposed mediator-based approach (triplet sink). All parameters in the simulations are the same for the two systems except that for the mediator approach the rate of homo-TTA was set to 0, and for the singlet sink approach the rate of hetero-TTA was set to 0. The concentration of annihilators was increased from 10^−3^ to 10^−2^ for the optimized singlet sink approach (singlet sink opt). Simulations were performed using a photon flux of 10^20^ photons per cm^2^ per s.

A common strategy to mitigate concentration quenching in solid-state TTA-UC is to introduce an emitter molecule at dopant concentrations. The emitter molecule does not take part in the upconversion process but functions as a singlet sink, receiving the energy from the S_1_ state of the annihilator *via* a Förster mechanism. It is interesting to compare the benefits of our triplet sink approach, with the conventional singlet sink approach. Fig. 8 shows the ratio of upconversion events and emitted photons per absorbed photons for both approaches. For the singlet sink approach, TTA can occur throughout the matrix, resulting in a higher yield of TTA events compared to the triplet sink approach. However, after the TTA event, the singlet sink approach relies on an energy transfer event, which limits the overall emission efficiency for two reasons. Firstly, the emission quantum yield of the annihilator is generally low, limiting the Förster distance, which causes a need to have relatively large dopant concentrations. Secondly, both the energy transfer step and the subsequent photon emission competes with Förster type resonance energy transfer to the sensitizer. This causes dual FRET loss channels and a need to have a significantly higher singlet sink compared to sensitizer concentration in order have a significant emission (Fig. 8). Thus, the singlet sink approach is much more sensitive for short circuiting due to energy transfer to the sensitizer.

In summary, kinetic Monte Carlo simulations can qualitatively reproduce the power dependence of upconversion in the solid state sensitized TTA system (binary blend). More importantly, in the ternary blend, simulations confirm that hetero-TTA outcompetes bulk homo-TTA at low excitation intensity conditions. At higher excitation fluences, the concentration of triplet mediator excitons increases, and with that the rate of homo-TTA is increased and disrupts hetero-TTA. Thus, the Monte Carlo simulations qualitatively reproduce the experimental results, strengthening the conclusion of a hetero-TTA process being active. Finally, by removing the ability of the mediator to reach the singlet state, the total number of TTA events does not actually decrease. Instead, the hetero-TTA events increase (Fig. S13a†) with increased yield of photon emission due to a reduced concentration induced quenching (Fig. S13b†), clearly indicating this is a very worthwhile strategy to pursue.

## Conclusions

This work proposes and examines a solution to the primary issue holding back solid-state TTA-UC, namely concentration-induced emission quenching within neat annihilator films. This is achieved by decoupling triplet energy diffusion and annihilation into two different molecules: a triplet mediator and an annihilator, respectively. The annihilator acts as a dilute triplet sink and emission center, and both experimental and computational results indicate that hetero-TTA between a triplet mediator and a triplet annihilator occurs to allow anti-stokes shifted emission. The upconverted emission profile of a sensitizer–mediator–annihilator blend at low excitation intensity conditions matches well with the emission profile of the annihilator. At higher photon fluxes, homo-TTA events (between two mediators) become dominant, thus indicating a change of mechanism from hetero-TTA (rubrene emission dominating) to homo-TTA (tetracene emission dominating) at higher exciton densities. Monte-Carlo based simulations capture the experimental observations qualitatively, corroborating the conclusions from the experimental work. The simulations also show that the benefits of our compared to the conventional singlet sink approach is that our approach is less sensitive against short circuiting the system *via* energy transfer back to the sensitizer.

The consequences of these findings are three-fold. Firstly, it provides further evidence to support previous studies in solution-based systems that suggest the existence of hetero-TTA UC channels.^34^ Secondly, it shows that Monte-Carlo based simulations capture essential features in solid state TTA-UC. Thirdly, and perhaps most importantly, these results show that exciton diffusion can indeed be decoupled from the exciton annihilation process, allowing for individual molecular optimization and for systems where concentration induced fluorescence quenching is not active.

We hope that this work will stimulate a new approach for achieving high performance solid state TTA-UC, and inspire along the guidelines set out here the development of ideal triplet mediators with very high singlet energies.

## Data availability

Raw data along with scripts used in simulations are stored at the Swedish National Data Service with the digital object identifier 10.5878/8sck-gs20 (https://doi.org/10.5878/8sck-gs20).

## Author contributions

AJC jointly devised the project, collected the experimental spectroscopic data, performed all synthesis, and wrote the manuscript. AMB performed the computational simulations and contributed with writing relevant sections. AD fabricated the binary and ternary blends, and with AM provided input in writing. VNG performed GIWAXS measurements. KB conceptualized the project and jointly devised it together with AJC, wrote the manuscript, and provided guidance throughout. All authors have given approval to the final version of the manuscript.

## Conflicts of interest

There are no conflicts to declare.

## Supplementary Material

SC-OLF-D4SC07004F-s001
