# Supplementary material for: Separating triplet exciton diffusion from triplet–triplet annihilation by the introduction of a mediator
Source: Chem Sci. 2024 Dec 9;16(3):1293–301. doi: 10.1039/d4sc07004f (PMC11639907; doi:10.1039/d4sc07004f)
Supplement: SC-016-D4SC07004F-s001 [file SC-016-D4SC07004F-s001.pdf]

Supplementary Information (SI) for Chemical Science.
This journal is © The Royal Society of Chemistry 2024

Supporting Information for:

Separating triplet exciton diffusion from triplet-triplet annihilation by the introduction of a mediator

Andrew J. Carrod,[a] Anton M. Berghuis,[b] Vishnu Nair Gopalakrishnan,[a] Andrew Monkman,[c] Andrew Danos,[c,d] Karl Börjesson[a*].

[a] University of Gothenburg, Department of Chemistry and Molecular Biology, Medicinaregatan 7B, 41390, Gothenburg, Sweden.

[b] Dutch Institute for Fundamental Energy Research, P.O. Box 6336, 5600 HH Eindhoven, The Netherlands

[c] Department of Physics, Durham University, South Road, Durham DH13LE, United Kingdom

[d] School of Physical and Chemical Sciences, Queen Mary University of London, London, E1 4NS, UK

Table of Contents

1. Methods and materials 2
   1.1 Synthesis 2
   1.2 Film Fabrication 2
   1.3 Photophysics 2
   1.4 Computational Simulations 3
2. Synthesis 5
3. NMR Spectra 8
4. Supplementary Figures 10
5. References 15

1. Methods and materials

1.1 Synthesis

Starting materials and solvents were purchased from Sigma-Aldrich Chemical Co., VWR, or Fisher Scientific, and they were used without further purification unless otherwise stated. All reactions were carried out using Schlenk techniques in oven-dried glassware, unless specifically stated. Solvents used for moisture- and oxygen-sensitive reactions were dried using an MBraun MB SPS-800 solvent purification system, or over molecular sieves (3Å). The sieves were obtained from commercial sources and dried at 300 °C for 24 h immediately before use. ^{1}H nuclear magnetic resonance (NMR) spectra were recorded on a Varian spectrometer at 400 MHz where ^{13}C NMR spectra were recorded at 101 MHz. Chemical shifts are given in parts per million using tetramethylsilane as an internal standard.

1.2 Film Fabrication

Films were deposited onto quartz or glass substrates that were firstly rinsed with acetone before drying under dry nitrogen. Substrates were loaded into a Kurt J Lesker Super Spectros 200 deposition system, equipped with Radak crucible-style thermal sources and QCM sensors to simultaneously monitor individual deposition rates for the annihilator, sensitizer, and mediator. Deposition rates for the samples were kept in the range of 1-2 Å s^{-1} and at pressures of 10^{-6} Torr or lower, with the ratio of rates determining the film composition. Deposition rates and specific source/material tooling factors were calibrated by confirming the nominal thickness of pure films, using a J.A. Woollam ellipsometer.

1.3 Photophysics

Solution based samples used for UV-Vis and emission spectroscopy were prepared in an Mbraun glove box having oxygen and water levels less than 1 ppm. Solvents were degassed with argon prior to use, then prepared and sealed in cuvettes with a cap and PTFE septum. Measurements were performed immediately after the preparation. UV-Visible spectra were recorded on a Perkin Elmer LAMBDA 950 instrument, whilst steady state emission spectra were measured on an Edinburgh Instruments FLS 1000 spectrofluorometer with a Xenon lamp as the excitation source and a double monochromator to select the wavelength. The photoluminescence quantum yield of PdTNP was determined from an indirect method with standard reference as recommend by IUPAC.[1] The quantum yield of the testing samples was calculated by the following Equation S1:

$$\Phi_x^i = \frac{F^i f_s n_i^2}{F^s f_i n_s^2} \Phi_f^s \qquad \text{S1}$$

Where Φ_x^i is the quantum yield of either upconversion or fluorescence. Φ_f^s is the known quantum yield of the standard. F^i and F^s are the integrated areas of the sample and standard emission spectra, respectively. f_i and f_s are the absorbance for the sample and reference, respectively. n_i and n_s are the refractive indices of the sample and reference solution, respectively. The excitation source (and wavelength of which) and detector were kept constant between reference and sample, therefore flux can be considered equal and ignored. Standard reference materials were selected by the excitation and emission spectrum range according to IUPAC recommendations, with $Ru(bpy)_3$ in water excited at 465 nm (QY = 0.04) used as a reference for Φ_{UC}^i.[1]

For Stern-Volmer measurements, degassed stock solutions of rubrene and tetracene were made inside a Mbraun glove box, and aliquots of which were added to cuvettes containing a pre diluted PdTNP solution at 5 µM. Measurements were carried out immediately after removal from the glovebox and the quenching effects were fitted based on equation S2 below.

$$\frac{\tau_0}{\tau} = \frac{I_0}{I} = 1 + k_{TET}\tau_0[A] \qquad \text{S2}$$

Solid samples prepared as films on quartz glass were sealed prior to use in photophysical measurements to ensure the absence of oxygen. First exposed to vacuum while being loaded into an Mbraun glove box, samples were sealed under inert atmosphere by affixing a second quartz plate over the film and sealing the edges using Loctite epoxy glue. Steady state emission of TTA-UC samples was measured on an Edinburgh Instruments FLS 1000 spectrofluorometer. Either a 450W Xe arc lamp, or a Coherent OBIS LX (690 nm) CW laser was used as an excitation source with variable power. Quantum yields were measured using the absolute method, utilising an integrating sphere purchased from Edinburgh instruments.

Luminescence decays were recorded either by time-correlated single photon counting (TCSPC) or Multichannel Scaling (MCS) on an Edinburgh FLS 1000 spectrofluorometer, using an MCP or PMT as detector. An Edinburgh instruments µF2 or pulsed diode laser (475 nm, 1 MHz) were used as excitation sources.

Transient absorption was measured on an Edinburgh Instruments LP 980 spectrometer, with a SpectraPhysics Nd:YAG laser (705 nm, pulse width ~7 ns) coupled to a Spectra-Physics primoscan optical parametric oscillator (OPO) as excitation. PMT (Hamamatsu R928) or image intensified CCD camera (ICCD, Andor DH320T-25F-03) detectors were used for recording transient kinetics or spectra, respectively.

1.4 Computational Simulations

For the simulations we used a time step size of 10 ps and a total number of 10^7 time steps. We stress that these Monte Carlo simulations are intended to get a qualitative understanding of the processes in the blends, and we therefore simplify the system by excluding the effect of disorder in the mediator material.

Binary Blend: Sensitizer and Mediator

We first introduce the binary blend. The mediator material is treated as a continuous material, while the sensitizer are discrete points in the continuous mediator material similar to the experimental case, the molar ratio of sensitizer to mediator is 1:100. The simulations are performed for a box of 50x50x50 nm. From the photon flux, the expected number of absorbed photons was calculated based on the molecular extinction coefficient (ε) of the sensitizer (PdTNP, 101,000 M^{-1} cm^{-1}), and the formed excitons were generated on the sensitizer molecules stochastically. These singlet excitons undergo fast intersystem crossing to a triplet state (τ_{ISC}). The formed triplets can then be transferred to the mediator material (τ_{SM}) but also have a small probability of reverse intersystem crossing back to the singlet state (τ_{RISC}). In the bulk material the triplets perform a diffusive (D_S) motion with a fixed hopping distance in a random direction within a sphere at each time step, where the hopping distance per time step is calculated from $D = \frac{ds^2}{6\,dT}$, where D is the diffusion constant, ds is the hopping distance per time step, and dT the time step in the simulation[4]. If two triplets are close to each other, they may undergo triplet-triplet annihilation (TTA) to a higher energy state. We model this process phenomenologically, and implemented this by stating that if two triplet states are within the Dexter radius of each other (r_{DexMM}) they annihilate at a time constant (τ_{TTAMM}). The upconversion process can result in a singlet

with a probability of 1/9, a higher lying triplet state with a probability of 3/9, or in a quintet state with a probability of 5/9[2,3]. If the TTA results in a quintet or triplet state, we consider this as a loss of one of the two triplet states. In case a singlet state is produced this singlet may decay radiatively with a time constant of τ_{RM}, this event is recorded as successful upconverted emission. A competing event to radiative decay is singlet fission into two triplet states (τ_{SFM}). During its lifetime, the singlet state also diffuses through the material (D_S). If the singlet exciton encounters a sensitizer site before it has decayed, it may get quenched by the sensitizer. The quenching is a Förster process with a rate of $k_Q = \frac{1}{\tau_{RM}}(\frac{r_F}{r})^6$ where r_F is the Förster radius, τ_{RM} is the intrinsic lifetime of the singlet exciton and r is the distance between the sensitizer and the singlet exciton on the mediator. The Förster distance was calculated using molar absorptivity spectra measured in solution and emission spectra and emission quantum yields measured in neat films. This quenching process is an important limiting factor for the efficient generation of upconverted emission.

Ternary Blend: Sensitizer, Mediator and Annihilator.

In the ternary blend, a third material is added to the blend that functions as an annihilator site that can trap a triplet state from the mediator (τ_{trap}) when the triplets get within the Dexter radius of the annihilator (r_{DexMA}). The molecular ratio of annihilator molecule to mediator molecules was set to 1:1000. Once a second triplet state reaches this annihilator site, hetero-TTA may occur (governed by the hetero-TTA (also sometimes referred to heterodyne) TTA rate (τ_{TTAMA}) and the dexter radius (r_{DexMA})), in a similar fashion to homo-TTA between two mediators. The large advantage of this path to upconversion is that the upconverted singlet state is energetically trapped at the annihilator molecule due to a potential difference of the excited singlet state in the annihilator material and the singlet states of the mediator. Therefore, the singlet will not diffuse and have a much lower chance to be quenched (it will only be quenched by the sensitizer if the annihilator site is very close to the sensitizer site) and have a higher probability to decay radiatively (τ_{RA}).

Determination of the Rates

The rates of the sensitizer (PdTNP) are based on both experimental values measured in this work and literature values,[2] and are stated in Table S1. The diffusion constant in the mediator (tetracene) was estimated to be one order of magnitude lower than in multi-crystalline tetracene.[3] The Dexter radius in both mediator and annihilator was fixed at 1 nm, on the order of the molecular spacing between the molecules.[4] Radiative lifetimes and the singlet fission time constants for both mediator and annihilator were fixed at 4 ns, which are optimistic values, resulting in an artificial higher photoluminescence quantum yield for both molecules.[5, 6] The reason for choosing these values is to be able to generate enough emission events in the simulations. This because generating many events at low excitation densities in the ternary blend results in extremely long computation times. By taking these optimistic rate constants we change the quantitative outcomes such as the quantum yield, but since the optimistic rates are chosen for both mediator and annihilator, the results are not affected qualitatively. The non-radiative decay rates of the mediator and annihilator triplet states are considered slow and set at 5 μs.[7] The trapping time of the triplet excited states on the annihilator is fast (0.1 ns) due to the lower energy of the annihilator states. The rates of the mediator-mediator TTA (homo-TTA) and the mediator-annihilator TTA (hetero-TTA) were varied in order to match the experimentally observed transition from mediator-mediator TTA-emission towards mediator-annihilator TTA emission at higher pump fluences.

Table S1: summarised photophysical parameters used in the simulation of TTA-UC in this work. In Figure 7, when simulating the difference between ideal singlet sink and triplet sink systems, then are the values for τ_{TTAMM} and τ_{TTAMA} set to infinitive for the triplet sink and singlet sink approach, respectively. Furthermore, Förster type energy transfer from the mediator to annihilator, r_{FMA}, where only set to a non-zero value when simulating the singlet sink approach (Figure 8).

Sensitizer		Mediator		Annihilator	
τ_{ISC}	0.1 ns	τ_{RM}	4 ns	τ_{RA}	4 ns
τ_{RISC}	100 ns	τ_{SFM}	4 ns	τ_{SFA}	4 ns
τ_{SM}	1 ns	τ_{nrM}	5 µs	τ_{nrA}	5 µs
τ_{MS}	10 ns	r_{DexMM}	1 nm	r_{DexMA}	1 nm
τ_{RS}	34 µs	τ_{TTAMM}	0.3 ns	τ_{TTAMA}	0.3 ns
r_F	3 nm	D_S	0.2×10^{-3} cm^2 s^{-1}	τ_{trap}	0.1 ns
ε	1×10^5 mol^{-1}cm^{-1}	D_T	2.7×10^{-5} cm^2 s^{-1}	$\tau_{release}$	100 ns
		r_{FMA}	1.6 nm		

2. Synthesis

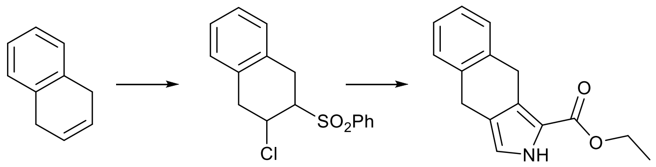

1 **2** **3**

Scheme S1: Synthetic route to Napthalene-based pyrrole **3**.

2-Chloro-1,2,3,4-tetrahydro-3-(phenylsulfonyl)-naphthalene (2)

Caution! A rapid exothermic reaction is likely to occur if initiation occurs after a large amount of thiophenol is added, care must be taken if undertaking this reaction. Around 0.5 mL of the total amount of thiophenol (4.09 g, 37.1 mmol) was added to a stirring suspension of *N*-chlorosuccinimide (5.08 g, 37.3 mmol) in dry dichloromethane (30 mL). The mixture was stirred for 10 mins until a deep orange colour appeared. Upon which, the solvent was kept at a gentle reflux by dropwise addition of the remaining thiophenol. After a further 1 hour of stirring, the precipitate was allowed to settle,before the orange PhSCl solution was added dropwise to a solution of **1** (4.0 g, 30.7 mmol) in DCM (25 mL). The resultant solution was stirred for 1 hour at room temperature, before being diluted to 100 mL with DCM and cooled to 0 °C. *m*-CPBA (18.0 g, 104.3 mmol) was added in one portion with rapid stirring. After stirring at 0 °C for 30 mins the reaction was warmed to room temperature for a further 30 mins, evaporated to dryness and the product recrystallised from EtOH (20 mL). This yielded a white precipitate (6.51 g, 24.6 mmol, 80 %). The spectral data match with reported values.[8] ^{1}H NMR ($CDCl_3$, 400 MHz): δ = 7.98-7.89 (m, 2H), 7.76-7.64 (m, 1H), 7.64-7.52 (m, 2H), 7.23-7.16 (m, 2H), 7.16-7.06 (m, 2H), 4.89-4.79 (m, 1 H), 3.76-3.66 (m, 1H), 3.54-3.40 (m, 1H), 3.35-3.24 (m, 1H), 3.20-3.03 (m, 2H). Mass was unable to be obtained due to instability.

4,9-Dihydro-2H-benzo[f]isoindole-1-carboxylic acid ethyl ester (3)

The title compound was prepared following a modified literature method.[8] **2** (2.00 g, 6.5 mmol) in dry THF (10 mL) was added dropwise to a solution of ^{t}BuOK (1.96 g, 15.7 mmol) with ethyl isocyanoacetate (0.89 g, 7.9 mmol) in dry THF (40 mL) at 0°C. The reaction was warmed to room temperature and then stirred for 18 hours. The solvent was removed, and the crude material dissolved in DCM (120 mL). The organic layer was washed with water (2 x 100 mL) and brine (1 x 100 mL) collected and dried over

Na_2SO_4. The crude material was recrystallized from EtOH and hexanes to yield **3** (400 mg, 1.7 mmol, 26 %). The spectral data match those reported. ^1H NMR ($CDCl_3$, 400 MHz): δ = 9.02-8.84 (br s, 1H), 7.35-7.30 (m, 1H), 7.22-7.16 (m, 2H), 6.84-6.80 (m, 1H), 4.35 (q, J = 7.2 Hz, 2H), 4.17 (s, 2H), 3.91 (s, 2H), 1.40 (t, J = 7.2 Hz, 3H). EI [M^+] Calc $C_{15}H_{15}NO_2$: 241.3, measured: 241.1.

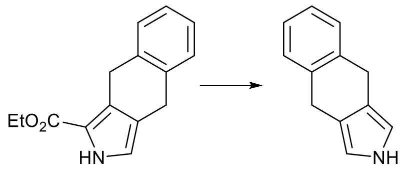

3 **NP**

Scheme S2: Deprotection of 9 to form the pyrrole **NP**.

4,11-dihydro-2H-naphtho[2,3-f]isoindole (NP)

3(400 mg, 1.7 mmol) and KOH (470 mg, 8.4 mmol) were suspended in ethylene glycol (20 mL) before purging the mixture thoroughly with argon. The mixture was heated to 170°C for 1 hour. The reaction was immediately cooled in an ice bath and diluted with DCM (100 mL). The organic layer was washed with water (2 x50 mL) and brine (1 x 50 mL) collected and dried over Na_2SO_4. The solvent was removed producing a brown solid (200 mg, 1.2 mmol, 71 %). GC analysis verified that no starting material was present, and a single peak was found for **NP**. The title compound was immediately used in a porphyrin synthesis without further purification due its instability.

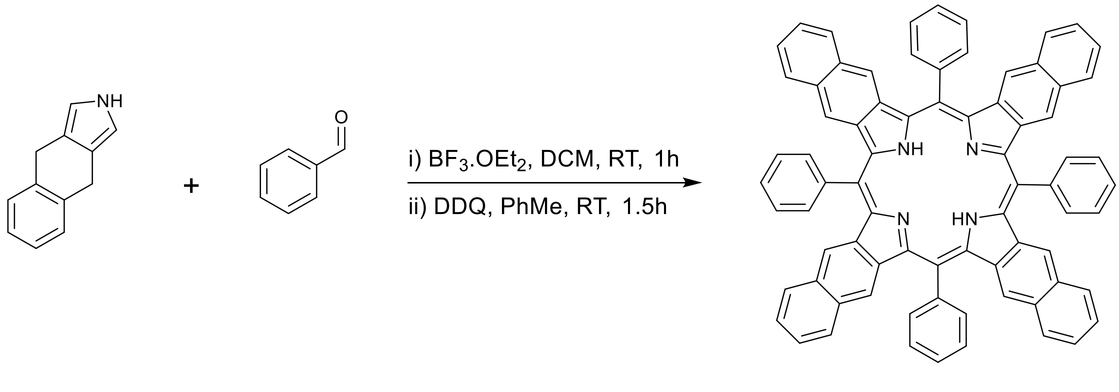

TNP

Scheme S3: Synthesis of **TNP** using modified Lindsey conditions.

tetraphenyltetranaphthoporphyrin (TNP)

In dry DCM (200 mL) thoroughly purged with nitrogen, N**P** (200 mg, 1.2 mmol) and benzaldehyde (127 mg, 1.2 mmol) were stirred in the dark, shielded by foil. Stirring was continued for 1 hour before addition of BF_3.Oet$_2$ (35 μL) in one portion. The reaction was stirred for a further hour before DDQ (1.4 g, 6.1 mmol) was added in degassed toluene (10 mL). The reaction was stirred for 90 mins, then added to 10% Na_2SO_3 (100 mL). The organic layer was separated, washed with 10% Na_2SO_3 (100 mL) and brine (100 mL). The aqueous phases were combined and washed with DCM (50 mL). The combined organic layers were dried over K_2CO_3, and the solvent removed under reduced pressure. The crude product was subjected to column chromatography on silica gel, using DCM as the eluent. The

combined fractions were dried and recrystallised in a mixture of 1:10, DCM:MeOH (11 mL). TNP precipitated over 2 days as green crystals (108 mg, 0.11 mmol, 37%). Spectroscopic analysis matches that reported in the literature.[8, 9] Protonation was carried out by addition of several drops of TFA to a solution of TNP in $CHCl_3$, before evaporation to dryness. ^{1}H NMR (Protonated form, $CDCl_3$, 400 MHz): δ = 8.68-8.55 (m, 8H), 8.11-7.86 (m, 20H), 7.75-7.68 (m, 8H), 7.53-7.47 (m, 8H). ^{13}C NMR (Protonated form, $CDCl_3$, 101 MHz): δ = 141.2, 137.4, 134.9, 133.1, 132.9, 129.6, 128.9, 128.1, 124.6, 122.4, 115.8. MALDI-MS [M$^+$] Calc $C_{76}H_{46}N_4$: 1014.3717, measured: 1014.3724. UV/vis for free base (toluene) λ_{max} (ε / 10^5 M^{-1} cm^{-1}) 500 (2.2), 675 (0.2), 726 (1.0), 749 (0.7).

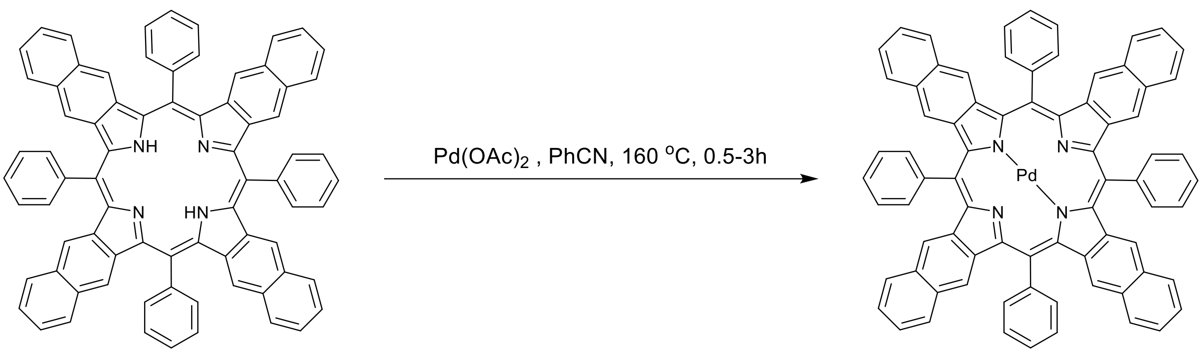

PdTNP

Scheme S4: Metalation of **NP** to yield **PdNP**.

palladium tetraphenyltetranaphthoporphyrin (PdTNP)

The palladium complex was obtained by heating of a mixture of porphyrin, excess $Pd(Oac)_2$ (2 equiv), in benzonitrile at 160 °C for 0.5–3 h (control by UV–vis spectroscopy), with subsequent filtration through a layer of silica (eluent: CH_2Cl_2) and evaporation of filtrate. The crude powder was recrystallised in a mixture of 1:10, DCM:MeCN (11 mL). NMR cannot be collected due to low solubility and high aggregation at relevant concentrations. MALDI-MS [M$^+$] Calc $C_{76}H_{44}N_4Pd$: 1118.2595, measured: 1118.2629. UV/vis (toluene) λ_{max} (ε / 10^5 M^{-1} cm^{-1}) 460 (1.0), 637 (0.1), 704 (1.5).

3. NMR Spectra

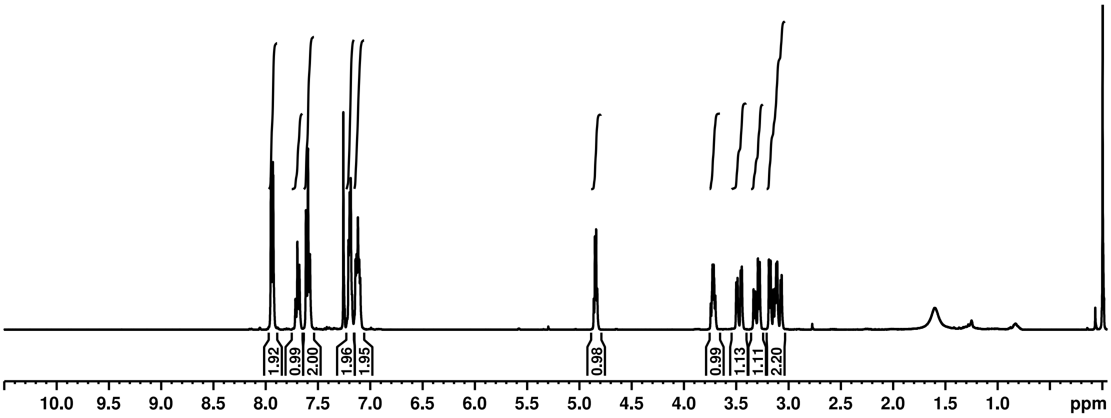

Figure S1: ^{1}H NMR in $CDCl_3$ of **2**.

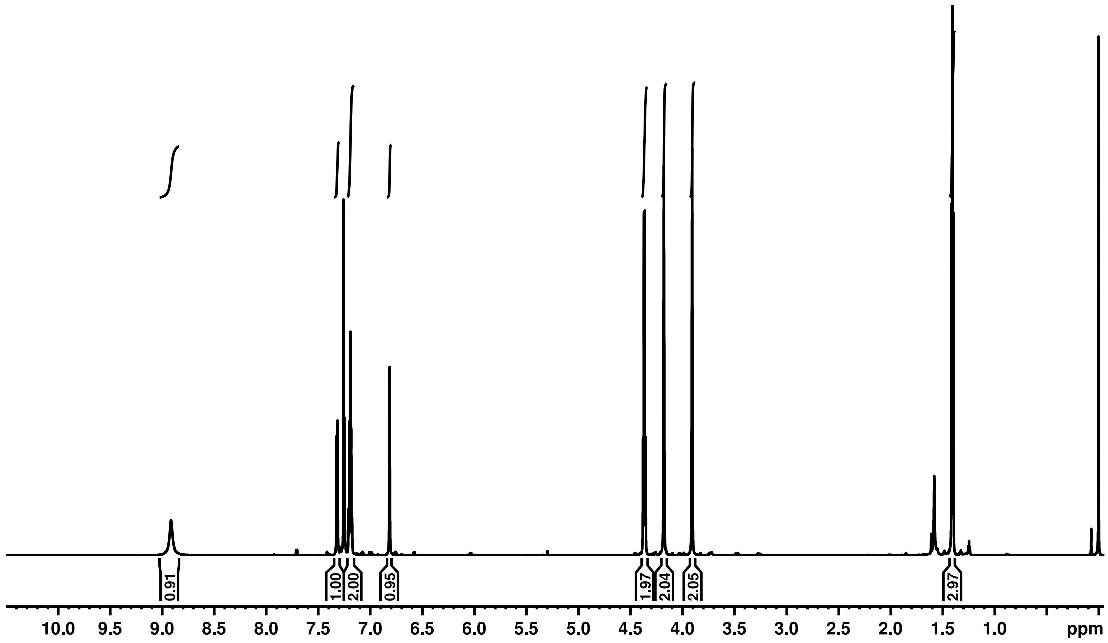

Figure S2: ^{1}H NMR in $CDCl_3$ of **3**.

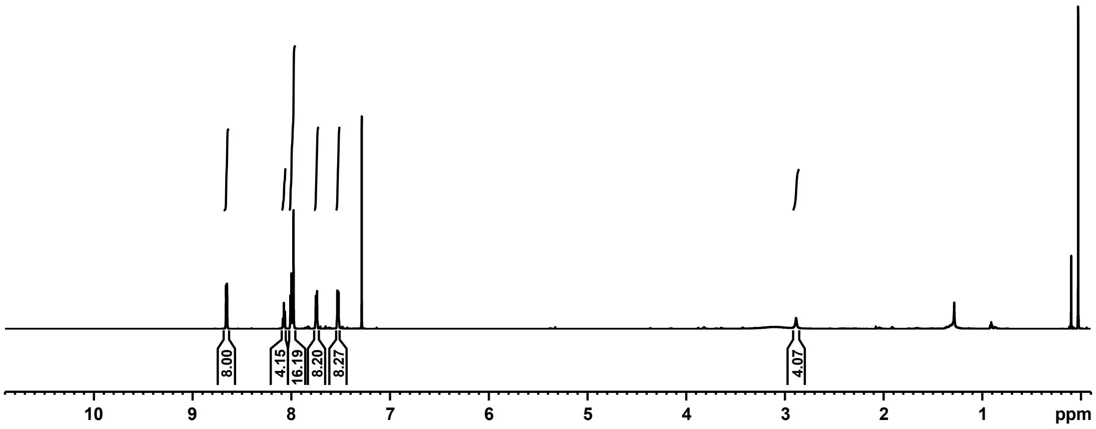

Figure S3: ^{1}H NMR in $CDCl_3$ of **TNP**.

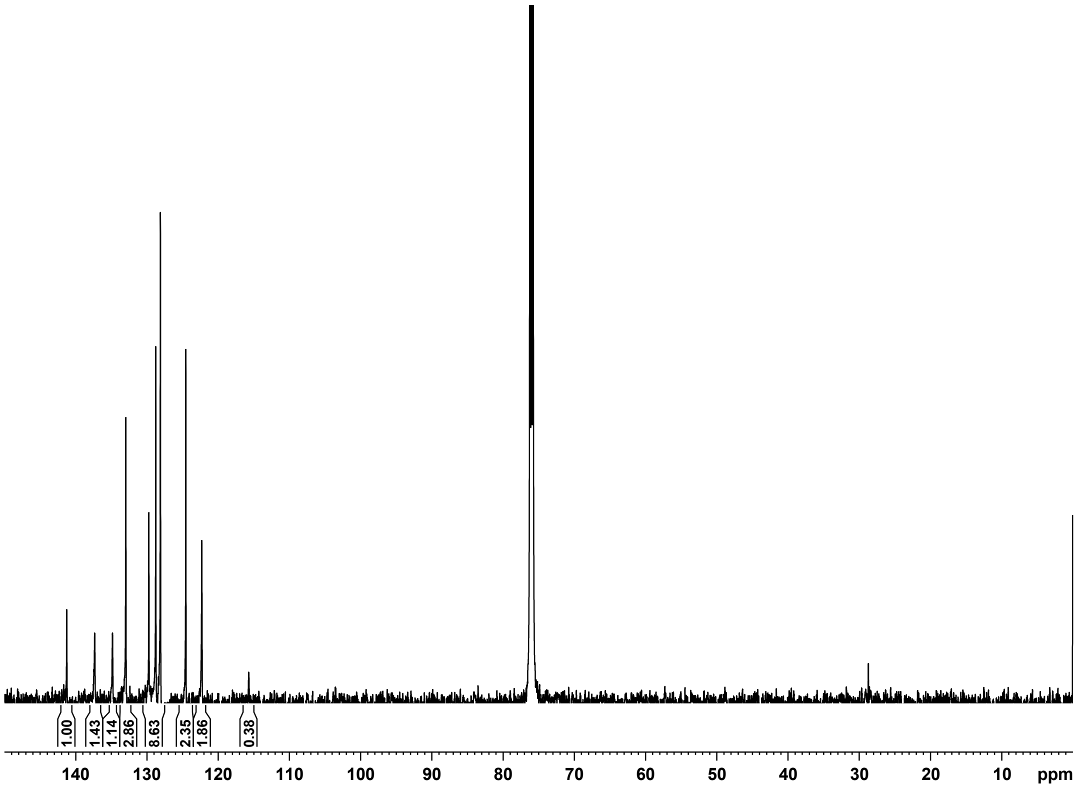

Figure S4. ^{13}C NMR in $CDCl_3$ of TNP.

4. Supplementary Figures

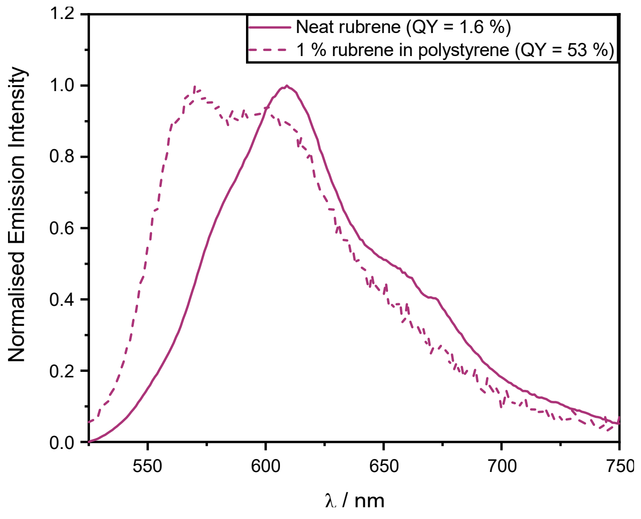

Figure S5: Emission spectra of a neat rubrene film (thermally evaporated, 100 nm, solid line) and a drop cast polymer film doped with rubrene, quantum yields measured for these films are indicated in the inset legend.

Table S2: Collated photophysical data for all porphyrins synthesised in this work.

**Compound**	**λ_{max} / nm** (**ε / 10^5 M^{-1} cm^{-1}**)	**Emission / nm**	**τ**	**Φ / %**	**k_r (k_{nr})**
TNP	500 (2.2), 726 (1.0)	755	4 ns	14	3.5×10^7 (2.2×10^8)
PdTNP	460 (1.0), 704 (1.2)	930	34 μs	3	1.1×10^3 (2.8×10^4)

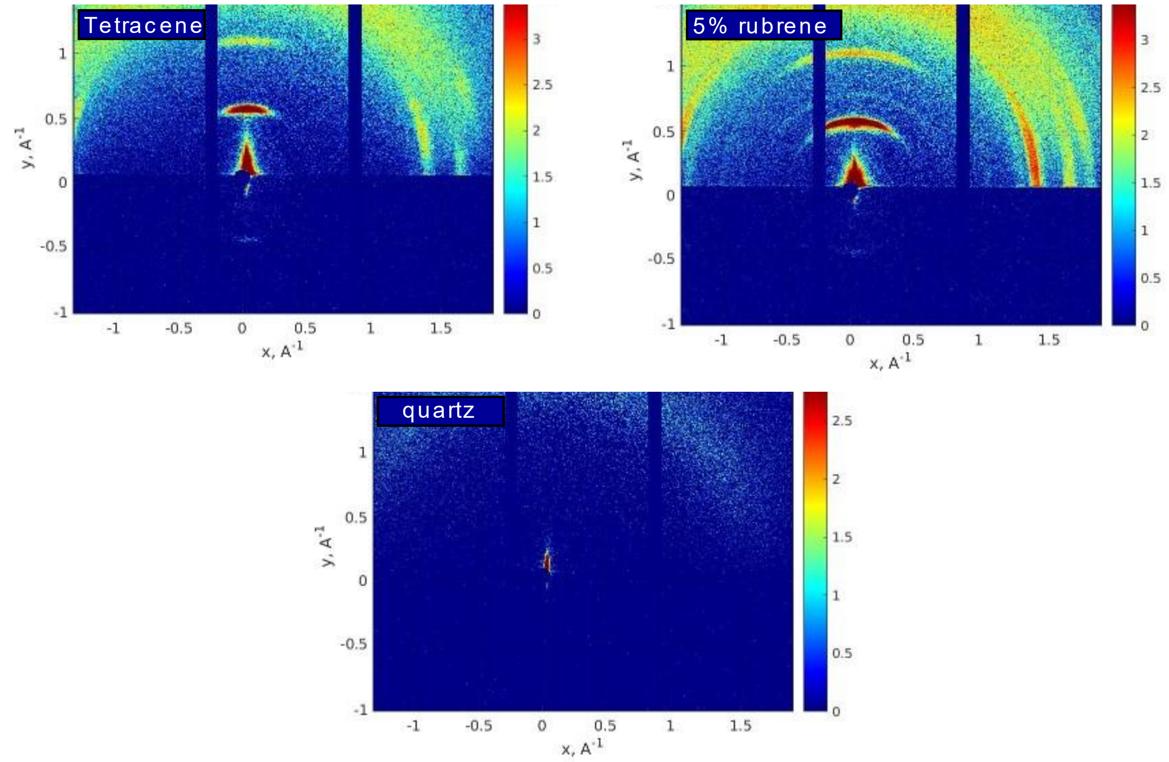

Figure S6: Reciprocal space maps measured by GIWAXS for thin films (100 nm) of tetracene and rubrene, and a blank quartz substrate. Measured with an incident angle of 0.2 Å.

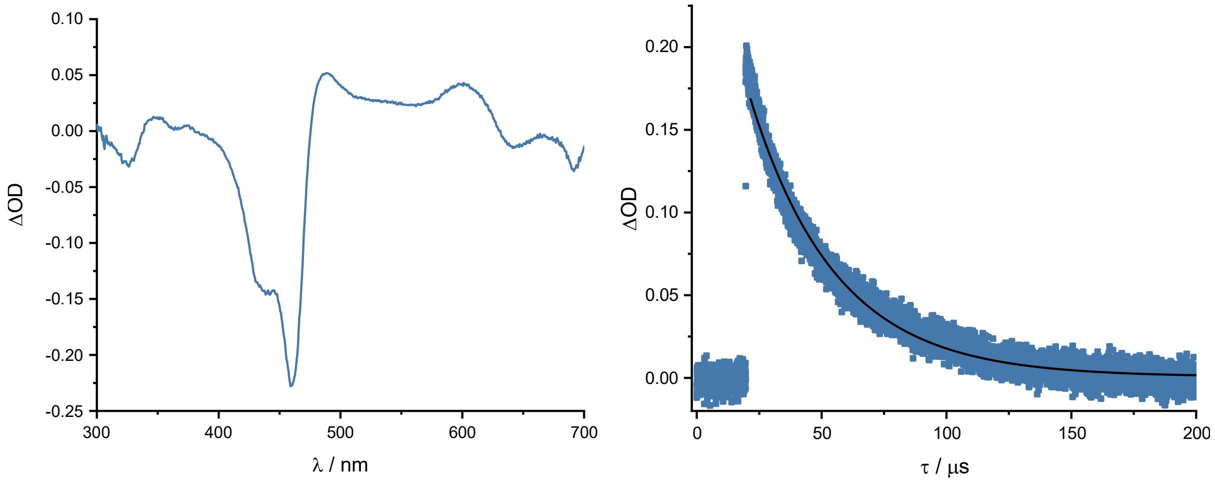

Figure S7: Left: Transient absorption spectrum taken at a 0 ns delay relative to the laser pulse for PdTNP (λ_{exc} = 705 nm , 5 μM, toluene). Right: Transient absorption decay taken for the excited state of PdTNP (600 nm, absorbance decay).

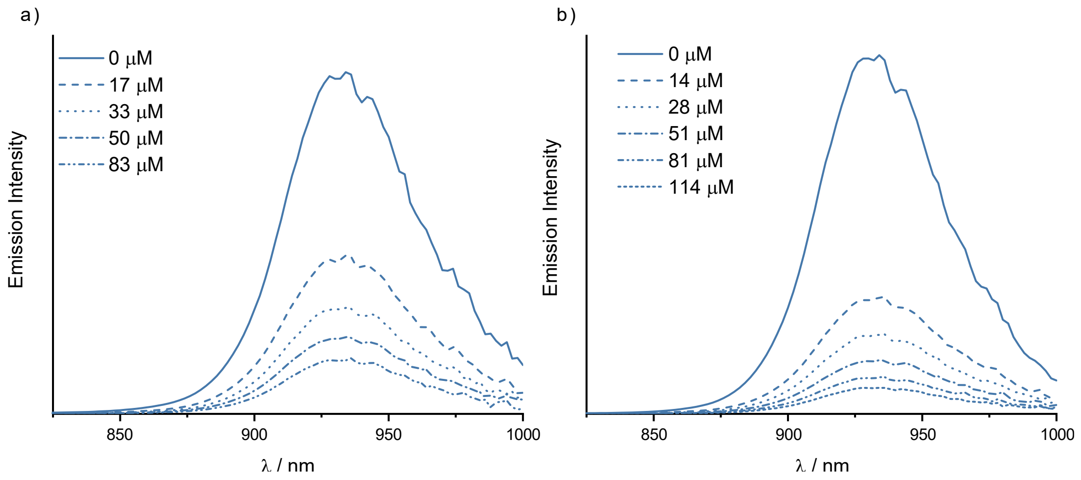

Figure S8: a) Emission spectra of PdTNP (λ_{exc} = 705 nm, 5 μM, toluene) solutions containing rubrene at the concentrations displayed. b) Emission spectra of PdTNP (λ_{exc} = 705 nm, 5 μM, toluene) solutions containing tetracene at the concentrations shown.

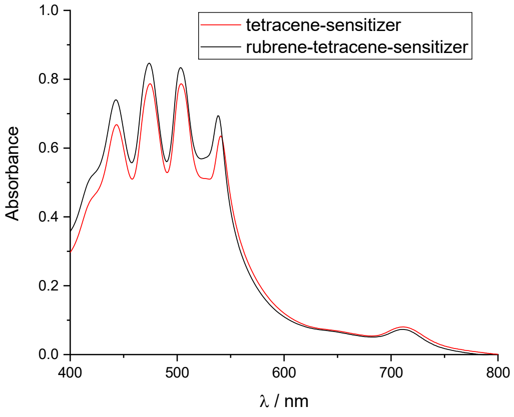

Figure S9. UV-Visible absorption spectra of the binary (tetracene-sensitizer) or ternary (sensitizer-tetracene-rubrene) blends.

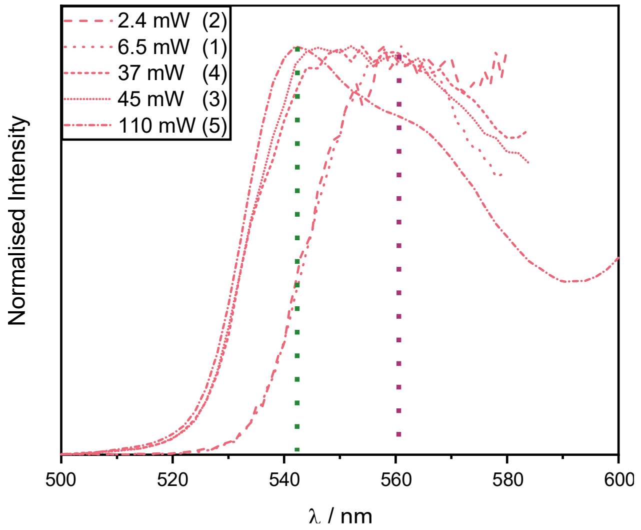

Figure S10: Repeats to ensure reversibility of the power dependent phenomena observed in the normalised emission spectra of PdTNP-tetracene-rubrene films (500 nm on quartz, 1% PdTNP and 1% rubrene). Incident power of the laser beam is indicated (λ = 690 nm, CW), vertical dashed lines indicate the positions of the transitions observed in these trimolecular films, with colours corresponding to the likely emitter species. Numbers within parentheses in the inset legend indicate the order in which the measurements were taken.

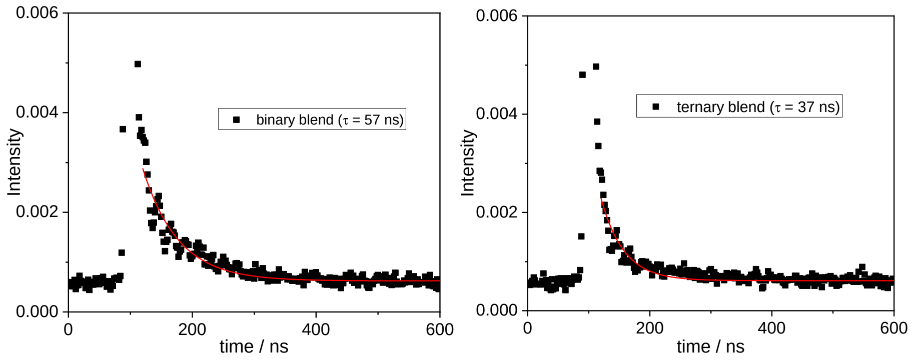

Figure S11: Upconverted emission from binary and ternary blended films. Excitation was performed using a SpectraPhysics Nd:YAG laser coupled to a Spectra-Physics primoscan optical parametric oscillator (OPO) set to 700 nm as the excitation source. The emission was recorded with the PMT set to 540 nm. The red curves show fits to a mono-exponential function (note that the initial signal is scattering from the laser and therefore not considered in the fit)

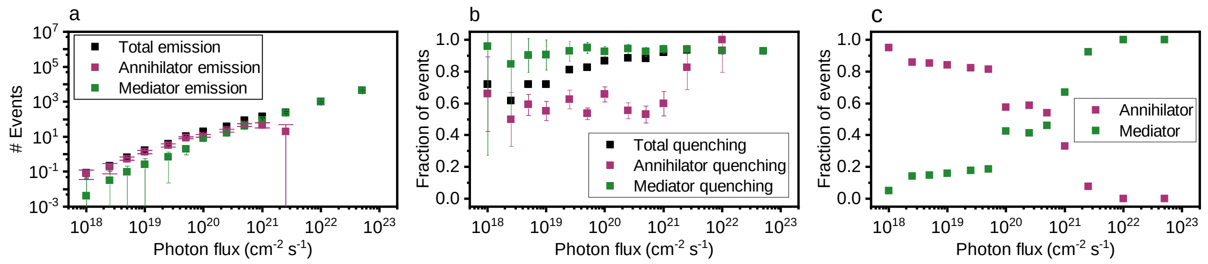

Figure S12: The effect of a ternary blend on Förster type energy transfer to the sensitizer. a) Total emission (black) and upconversion events from homoTTA (green) and heteroTTA (purple) mechanisms. b) Fraction of upconversion events that are quenched by energy transfer to the sensitizer. c) Fraction of upconversion events occurring within the mediator or annihilator as a function of photon flux. Parameters used in the simulations are found in Table S3.

Table S3: Parameters used in the simulations shown in Figure S10.

Sensitizer		Mediator		Annihilator	
τ_{ISC}	0.1 ns	τ_{RM}	4 ns	τ_{RA}	4 ns
τ_{RISC}	100 ns	τ_{SFM}	4 ns	τ_{SFA}	4 ns
τ_{SM}	1 ns	τ_{nrM}	5us	τ_{nrA}	5us
τ_{MS}	10 ns	r_{DexMM}	3 nm	r_{DexMA}	3 nm
τ_{RS}	34 µs	τ_{TTAMM}	0.3 ns	τ_{TTAMA}	0.3 ns
r_F	3 nm	D_S	0.2 x 10^{-3} cm^2 s^{-1}	τ_{trap}	0.1 ns
ε	1 x 10^5 $mol^{-1}cm^{-1}$	D_T	2.7 x 10^{-5} cm^2 s^{-1}	$\tau_{release}$	100 ns

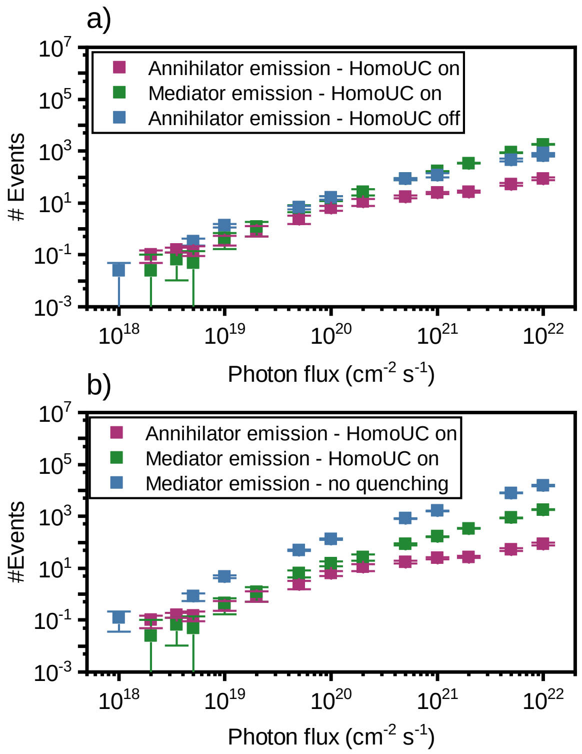

Figure S13: a) This figure shows two simulations. purple and green squares show UC in a ternary blend with UC in the mediator being TTA-active. The blue squares show UC events where the mediator TTA is inactive. The total number of upconversion events are the same for these two experiments. b) The same as for (a), except that the fluorescence quantum yield of the annihilator has been increased due to no concentration induced fluorescence quenching being ideally active in a high bandgap matrix.

5. References

1. A. M. Brouwer, *Pure Appl. Chem.*, 2011, **83**, 2213-2228.
2. A. Harriman, *J. Chem. Soc., Faraday Trans. 2*, 1981, **77**, 1281-1291.
3. A. M. Berghuis, T. V. Raziman, A. Halpin, S. Wang, A. G. Curto and J. G. Rivas, *J. Phys. Chem. Lett.*, 2021, **12**, 1360-1366.
4. B. Daiber, K. van den Hoven, M. H. Futscher and B. Ehrler, *ACS Energy Lett.*, 2021, **6**, 2800-2808.
5. J. J. Burdett and C. J. Bardeen, *Acc. Chem. Res.*, 2013, **46**, 1312-1320.
6. J. J. Burdett, A. M. Müller, D. Gosztola and C. J. Bardeen, *J. Chem. Phys.*, 2010, **133**.
7. M. Pope, N. E. Geacintov, D. Saperstein and F. Vogel, *J. Lumin.*, 1970, **1-2**, 224-230.
8. O. S. Finikova, S. E. Aleshchenkov, R. P. Briñas, A. V. Cheprakov, P. J. Carroll and S. A. Vinogradov, *J. Org. Chem.*, 2005, **70**, 4617-4628.
9. J. R. Sommer, A. H. Shelton, A. Parthasarathy, I. Ghiviriga, J. R. Reynolds and K. S. Schanze, *Chem. Mater.*, 2011, **23**, 5296-5304.
